# SFTSV infection in rodents and their ectoparasitic chiggers

**DOI:** 10.1371/journal.pntd.0010698

**Published:** 2022-08-29

**Authors:** Xiao-Lan Gu, Wen-Qing Su, Chuan-Min Zhou, Li-Zhu Fang, Ke Zhu, Dong-Qiang Ma, Fa-Chun Jiang, Ze-Min Li, Dan Li, Shu-Hui Duan, Qiu-Ming Peng, Rui Wang, Yuan Jiang, Hui-Ju Han, Xue-Jie Yu

**Affiliations:** 1 State Key Laboratory of Virology, School of Public Health, Wuhan University, Wuhan, China; 2 Qingdao West Coast New District Center for Disease Control and Prevention, Qingdao, China; 3 Qingdao Municipal Center for Disease Control and Prevention, Qingdao, China; 4 School of Public Health, Shandong First Medical University & Shandong Academy of Medical Sciences, Ji’nan, China; National Institute of Infectious Disease, JAPAN

## Abstract

SFTSV, a tick-borne bunyavirus causing a severe hemorrhagic fever termed as severe fever with thrombocytopenia syndrome (SFTS). To evaluate the potential role of rodents and its ectoparasitic chiggers in the transmission of SFTSV, we collected wild rodents and chiggers on their bodies from a rural area in Qingdao City, Shandong Province, China in September 2020. PCR amplification of the M and L segments of SFTSV showed that 32.3% (10/31) of rodents and 0.2% (1/564) of chiggers (*Leptotrombidium deliense*) from the rodents were positive to SFTSV. Our results suggested that rodents and chiggers may play an important role in the transmission of SFTSV, although the efficiency of chiggers to transmit SFTSV needs to be further investigated experimentally.

## Author summary

Severe fever with thrombocytopenia syndrome (SFTS), an emerging hemorrhagic fever in Asia, is caused by SFTSV, a tick-borne bunyavirus. In this study, we analyzed the prevalence of SFTSV in rodents and their ectoparasite chigger mites (*Leptotrombidium delicense*) with RT-PCR. RT-PCR results showed that one third (32.3%, 10/31) rodents and one chigger (0.2%, 1/564) was positive to SFTSV. Our study suggested that rodents and chiggers may play an important role in the ecology of SFTSV.

## Introduction

Severe fever with thrombocytopenia syndrome (SFTS) is an emerging hemorrhagic fever disease, caused by a tick-borne bunyavirus SFTSV (*Dabie bandavirus*) [1–3]. SFTS cases had non-specific clinical manifestations, such as fever, fatigue, myalgia, and gastrointestinal symptoms [1], which can be easily misdiagnosed as other febrile disease such as scrub typhus and hemorrhagic fever with renal syndrome (caused by hantaviruses) with similar clinical manifestation and increased the risk of death [4,5]. The case fatality rate of SFTS ranges from 3% to 30% among patients in different countries in East Asia [1,6–8], and lack of specific anti-SFTSV medicine or vaccines to combat the SFTSV. Since the disease was reported in China in 2011, SFTS cases has been confirmed by isolation of SFTSV or detection of SFTSV RNA in Japan, South Korea, Myanmar, Vietnam, and Thailand [1,6,9–12]. As it causes a severe disease and its endemic area continues to expand, SFTSV had been included in a list of priority pathogens with the potential to generate an international public health emergency by the World Health Organization in 2015 [13]. SFTS is typically considered as a tick-borne disease, and the virus has been detected in many tick species and laboratory transmitted in several tick species [2,14], but SFTSV can also be transmitted through mouth mucosa or conjunctiva to cause person-to-person or animal-to-human infection [15–17]. SFTSV genome had been detected from ticks in China, South Korea, and Japan [2,18–21].

Mites of the family Trombiculidae (known as chigger mites, sand mites, and harvest mites) are a large group of medical-relevant arthropods lived in moist soil covered with vegetation [22,23]. The word “chigger” refers to the larval stage of trombiculid mites among their life cycle (egg, larva, nymph, adult), as it is the only parasitic stage and feeds on decomposed tissues and lymph fluid of the hosts [24]. Chiggers have a worldwide distribution and infest a wide range of animals, including vertebrate and non-vertebrate, particularly rodents [25]. The most important medical significance of chigger is as the vector of *Orientia tsutsugamushi*, which causes scrub typhus [26]. Only a minority of chiggers can spread scrub typhus effectively, and they belong to the genus *Leptotrombidium* [27]. *Leptotrombidium scutellare* is ranked only second to *L*. *deliense* as the important vector of scrub typhus in China [28].

In recent years, with more and more reports of co-infection of SFTSV and *O*. *tsutsugamushi* [12,29–31], as well as, SFTS and scrub typhus had overlapped epidemic seasons [30,32,33], it is suspected that chiggers might play a role in the transmission of SFTSV. In addition, a Chinese literature reported that SFTSV was detected by PCR in mites (*Laelaps echidninus*, family Laelapidae and *L*. *scutellare*, family Trombiculidae) collected from rodents and goats in Jiangsu Province [34], which further deepened the suspicion of chigger’s role in transmission of SFTSV. However, only a single study reporting PCR detection of SFTSV in chiggers without isolation of the virus, the role of chiggers in transmission of SFTSV is still not clear because we do not know whether the PCR-detected SFTSV was degraded or inactivated viral genome in mites in the previous study. To understand the infection of SFTSV in chiggers, we used molecular method to analysis SFTSV infection in rodents and chiggers collected on the rodents from Qingdao City of Shandong Province, China.

## Material and methods

### Ethics statement

Collection of rodents was approved by the ethics committee of Medical School, Wuhan University (WHU2020-YF0023) and carried out in accordance with Wuhan University Guidelines on the Care and Use of Laboratory Animals.

### Sampling

In September 2020, rodents and insectivore were captured from Qingdao City of Shandong Province in Eastern China. Rodents and insectivore were captured with snap traps baited with peanuts in wine, which were set bordering crop field each night, and checked the next morning. The captured animals were placed individually in transparent sealing bags labeled with the sampling number, date, and site of collection, and were taken back to the laboratory of a local center for disease control and prevention to collect rodent tissue and ectoparasites. Under a digital microscope, mites from animals and sealing bags were carefully collected using the sharp end of an acne needle. Mites of same species from a host were pooled into microcentrifuge tubes according to morphology [35]. Tick was not found on any rodents. Samples were placed into a bucket of dry ice for transportation, and once arrived at the laboratory, samples were immediately stored at -80°C.

### Nucleic acid extraction and molecular detection

Mites in each pool were rinsed with sterile phosphate buffer saline (PBS) and homogenized with steel beads before nucleic acid extraction. DNA and RNA were extracted from each pool of mites and each spleen sample of rodents using the AllPrep DNA/RNA Mini Kit (QIAGEN, Hilden, Germany) and were stored at -80°C.

The RNA template was reverse transcribed into cDNA with RT First Strand cDNA Synthesis Kit (Servicebio, Wuhan, China). Nested polymerase chain reaction (PCR) targeting L and M segments of the SFTSV was performed. Both primary and nested PCRs were performed at 94°C for 5 min, followed by 35 cycles at 94°C for 30 s, 55°C for 30 s, 72°C for 30–60 s, and a final extension at 72°C for 10 min. The species of mites and small animals were identified by PCR amplification of the barcording region of the mitochondrial cytochrome c oxidase subunit I gene (*COI*) as described previously [36,37]. Primers used in this study were shown in Table 1. Each PCR reaction included a negative control with diethyl pyrocarbonate (DEPC)-treated ddH_2_O instead of cDNA as the template. Nucleic acid extraction, PCR preparation, and agarose gel electrophoresis were performed in separate rooms to avoid cross-contamination.

**Table 1:** Polymerase chain reaction primers used in this study.

Target genes	Primers	Primer Sequence (5’-3’)	Size (bp)	Reference
SFTSV L segment	SFTSV-L-F1	AATGATGCCAAGAAGTGGAAT	855	This study
	SFTSV-L-R1	ATGTAAGCATAGTCCTAGAAGC		
	SFTSV-L-F2	CCACAGATTCATTTGGGCT	367	
	SFTSV-L-R2	ATCATGATCGCTGAGTCGTC		
SFTSV M segment	SFTSV-M-F1	TCTGCAGTTCAGACTCAGGGA	761	[38]
	SFTSV-M-R1	GACGTGTATTGCTGTTTTCCC		
	SFTSV-M-F2	TGTTGCTTGTCAGCCTATGAC	674	
	SFTSV-M-R2	CAACCAATGATCCTGAGTGGA		
Mites *COI*	COI-F	GGTCAACAAATCATAAAGATATTGG	710	[36]
	COI-R	TAAACTTCAGGGTGACCAAAAAATCA		
Small mammals *COI*	COI-F	ACTTCTGGGTGTCCAAAGAATCA	750	[37]
	COI-R	CCTACTCRGCCATTTTACCTATG		

*COI*: mitochondrial cytochrome c oxidase subunit I gene

PCR products were analyzed in a 1.5% agarose gel containing 4S GelRed (Sangon, Shanghai, China) and checked under an ultraviolet light. PCR products with expected size were excised and purified using Gel Extraction Kit (Tsingke, Beijing, China). Purified DNA was inserted into pMD 19-T vector (TaKaRa, Dalian, China) and transformed into *Escherichia coli* DH5α competent cells for cloning and sequencing (Sangon, Shanghai, China).

### Phylogenetic analysis

Chromatograms were checked with Chromas (Technelysium, Tewantin, QLD, Australia) to ensure the accuracy of sequencing. The obtained sequences were identified by comparing with sequences in GenBank with BLAST programs (https://blast.ncbi.nlm.nih.gov/Blast.cgi). The sequence alignment and editing were performed with the MEGA 7.0 software (http://www.megasoftware.net). Phylogenetic trees were constructed based on Maximum Likelihood (ML) method with the Kimura 2-parameter model using the MEGA 7.0 software, bootstrap values were calculated with 1,000 replicates.

## Results

### Small mammal and mite collection

**Fig 1:**
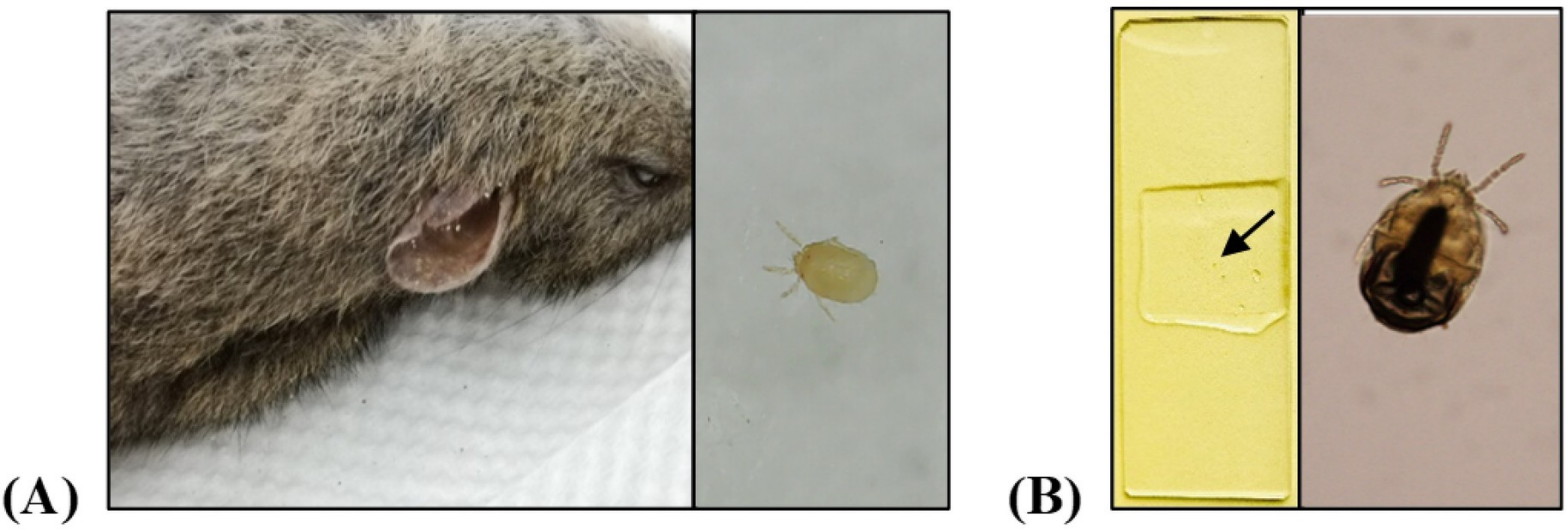
Photograph of chiggers collected from small mammals. (A) A *Tscherskia triton* infested by chiggers in the external ear canal (left) and a chigger photographed with digital microscope (right). (B) A chigger was fixed on the microscope slide (left, as indicated by the arrow) and photographed with an Olympus microscope (right).

Thirty rodents (12 *Apodemus agrarius*, 11 *Tscherskia triton*, 6 *Mus musculus* and 1 *Cricetulus griseus*) and one shrew (*Crocidura lasiura*) were captured from rural area in Huangdao District, Qingdao City of Shandong Province, China. A total of 564 mites were collected from small mammals (Fig 1). Mites from each animal were pooled together (average 23 mites/pool, ranging from 9 to 50 mites/pool) to analyze SFTSV. All mites belonged to the family Laelapidae and family Trombiculidae based on *COI* gene sequence (Table 2).

**Table 2:** Prevalence of SFTSV in rodents, insectivore and mites.

Host species	No. of host animals (%)	SFTSV positive rate (%)	No. of mites (%)	No. of mite pools	No. of mite pool positive to SFTSV
*Apodemus agrarius*	12 (38.7)	4 (33.3)	107 (19.0)	8 (2 Laelapidae; 6 Trombiculidae)	0
*Tscherskia triton*	11 (35.5)	4 (36.4)	429 (76.1)	15 (Trombiculidae)	1
*Mus musculus*	6 (19.4)	2 (33.3)	9 (1.6)	0	0
*Cricetulus griseus*	1 (3.2)	0	10 (1.8)	1 (Trombiculidae)	0
*Crocidura lasiura*	1 (3.2)	0	9 (1.6)	1 (Laelapidae)	0
Total	31	10 (32.3)	564	25 (3 Laelapidae; 22 Trombiculidae)	1

### Identification of SFTSV in rodents and mites

The SFTSV M segments were amplified from 10 (32.3%, 10/31) rodents, including 4 *Apodemus agrarius*, 4 *Tscherskia triton*, and 2 *Mus musculus*, but none in shrew. One pool of chiggers (*L*. *deliense*, n = 23) from a greater long-tailed hamster (*Tscherskia triton*) was also positive to SFTSV by the M and L segments.

### Phylogenetic analysis

**Fig 2:**
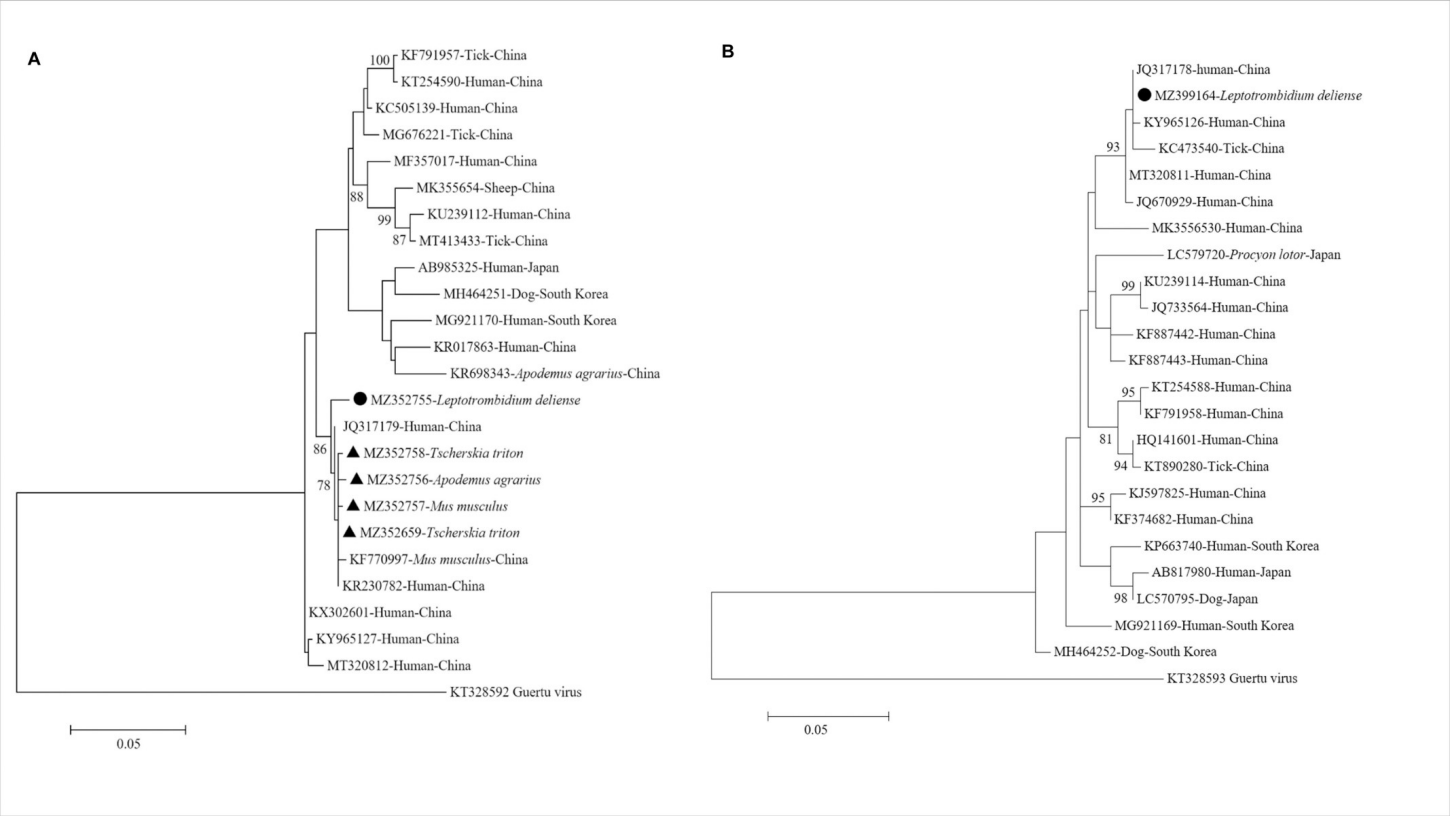
Phylogenetic analysis based on the partial nucleotide sequences of SFTSV M and L segments. (A) M segment (638bp). (B) L segment (327bp). The SFTSV sequences obtained in this study were marked by triangles (from rodents) and a circle (from chiggers). The phylogenetic trees were generated with Maximum Likelihood method by the MEGA 7.0 software (1,000 bootstrap replicates). Bootstrap value >75% was shown at nodes. The Guertu virus, belonging to the genus *Phlebovirus* of the family *Phenuiviridae*, was used as outgroup. Scale bars indicated substitutions per site. The reference sequences were named as: GenBank accession number-host-country.

Phylogenetic analysis showed that the partial SFTSV M segment obtained from rodents in this study shared 99.4%-100.0% nucleotide homology with each other, and were closely related to those obtained from a *Mus musculus* (KF770997) in Shandong Province and a SFTSV patient (KR230782) in Jiangsu Province, China. The partial SFTSV M segment sequence obtained from *L*. *deliense* in this study exhibited 98.6%-98.9% nucleotide identity with the SFTSV sequences from rodents, and shared 99.1% nucleotide identity with the sequence from a SFTS patient (JQ317179) in Jiangsu Province, China. Coincidentally, the SFTSV positive chiggers were collected from a long-tailed hamster that was also positive to SFTSV, but the sequences of SFTSV M segment from the chiggers and the hamster were different (98.9% homology), indicating the SFTSV in the chigger was not from the hamster. The M segment obtained from hamster had 99.8% nucleotide identity with SFTS patient (JQ317179) from Jiangsu Province, China (Fig 2A). The partial SFTSV L segment detected in *L*. *deliense* shared 100% nucleotide identity to the sequence from a SFTS patient (JQ317178) in Jiangsu Province, China (Fig 2B). Sequences of this study were deposited in GenBank with accession numbers: MZ352755-MZ352765, MZ399164, MZ389783-MZ389787, MZ389209-MZ389211.

## Discussion

In this study, we found that a high prevalence of SFTSV in rodents (32.3%) from rural area in Qingdao City, Shandong Province, China by PCR amplification. The positive rodents included *Apodemus agrarius*, *Tscherskia triton*, and *Mus musculus*. PCR preparation and agarose gel electrophoresis were performed in separate rooms, which could eliminate cross-contamination. Furthermore, the SFTSV sequences from different animals varied, confirmed that the STSV sequences obtained in this study were genuine and were not from contamination. To our knowledge, the positive rate of SFTSV in the rodents was higher than any other animal species investigated previously [38–41], suggesting rodents may be the major animal hosts of SFTSV. In a previous study we demonstrated that rodents were positive to SFTSV at a low positive rate (0.7%) [38]. The sequences of M segment for SFTSV obtained from rodents and chigger in this study also closely related to corresponding sequences from *Mus musculus* in previous study (KF770997, Fig 2A). Both our previous study and this study have captured rodents in rural areas of Huangdao District of Qingdao City, but at different seasons and years. The rodents in the previous study were captured from January to August in 2013 and the rodents in this study were captured in September, 2020. It was reported that density of field rodents had a peak in the third quarter (July to September) in Qingdao City [42]. The optimum temperature for the development and reproduction of *L*. *deliense* was 18–28°C [43], and the average temperature in Qingdao was 22.3°C in September, 2020, according to the Meteorological Bureau of Shandong Province [44]. As the only parasitic stage among its life cycle, chiggers parasitize mainly on rodents and other small mammals [45]. Collectively, high density of rodents and chiggers might contribute to the high prevalence of SFTSV in rodents in this study. The seasonal variation of SFTSV positive rate in rodents need to be further studied.

We also demonstrated that a pool of chiggers (*L*. *deliense*) fed on an infected hamster was positive to SFTSV with low infection rate (0.2%). The SFTSV sequence from the chiggers was different from the SFTSV sequence from the hamster host, indicating that the chigger might obtain SFTSV from another animal or vertically. We used PCR to detect the host *COI* gene to demonstrate whether host DNA existed in the chigger’s digestion system when we extracted chiggers’ DNA. We could not amplify the host *COI* gene in the extracted chiggers’ nucleic acid, indicating that the host nucleic acid had been degraded in the chigger’s midgut. By analogy, any SFTSV in the chiggers’ meal (liquefied host tissues) should also been degraded in the midgut. Thus, the SFTSV RNA detected in the chiggers must be from SFTSV existing in the chigger body, but not degraded SFTSV RNA in the liquefied host tissue in the meal. This result indicated that SFTSV survived in the body of chiggers. These results suggested that chigger may play a role in transmission of SFTSV among rodents and to humans. In total we analyzed 564 chiggers, with 179 from 10 infected rodents, only one chigger was positive to SFTSV. However, the extreme small body size (0.4 mm) of chigger may limit the amount of SFTSV RNA in a chigger and reduce the sensitivity of PCR for detection SFTSV, which may make us underestimate the positive rate of SFTSV in chigger. In order to detect SFTSV, we pooled chiggers together, which could further reduce the sensitivity of PCR detection of SFTSV in chiggers. Therefore, the role of chigger in transmission of SFTSV needs to be further experimentally verified.

It is worth noting that the prevalence of SFTSV and viral loads in the major vector, *Haemaphysalis longicornis*, are very limited. The prevalence of SFTSV in *H*. *longicornis* in China and South Korea were 0.2% (8/3,300) and 0.5% (55/11,856), respectively [2,46]. It was reported that SFTSV RNA was detected in 6.0% (233/3,898) individual ticks collected from the Deogyusan National Park, including 5.9% (218/3,671) *H*. *longicornis* and 7.7% (15/194) *H*. *flava*, but none (0/32) in *Ixodes nipponensis* in the Republic of Korea [47]. Another study in the Republic of Korea reported SFTSV was detected in 4.77% (48/1,006) *H*. *longicornis*, 1.15% (5/436) *H*. *flava*, and 20% (1/5) in *A*. *testudinarium*, also none (0/23) in *I*. *nipponensis*, collected from vegetation in five national parks [48]. These studies indicated *H*. *longicornis* and *H*. *flava* were main vectors for SFTSV. Ticks were not identified on the rodents in this study. A possible explanation is that ticks had abandoned dead hosts and another possibility is that the active season of ticks has passed when the rodents were collected. The peak season of SFTS in Shandong Province is May to August [49].

In this study, we only used M segment for phylogenetic analysis of chiggers and hosts. We also tried to amplified S segment and L segment of SFTSV, but only got 2 positive results for SFTSV L segment of rodents. We got positive result from chiggers with primers from a conserved region of SFTSV L segment. However, the L segment of SFTSV obtained from rodents and chigger were not overlapped, we could not compare the homology between them.

In conclusion, SFTSV RNA was identified in a high proportion of rodents and in a low proportion of chiggers (*L*. *deliense*). Our results indicated that rodents may play an important role in transmission of SFTSV and the role of chiggers in transmission of SFTSV need to be further investigated.
